# Outcome of patients with early arthritis without rheumatoid factor and ACPA and predictors of rheumatoid arthritis in the ESPOIR cohort

**DOI:** 10.1186/s13075-019-1909-8

**Published:** 2019-06-06

**Authors:** Gaël Mouterde, Nathalie Rincheval, Cédric Lukas, Claire Daien, Alain Saraux, Philippe Dieudé, Jacques Morel, Bernard Combe

**Affiliations:** 1 Rheumatology Department, CHU Montpellier, Univ Montpellier, Montpellier, France; 2 EA 2415, Montpellier, France; 3 Biostatistics, Epidemiology Unit, Montpellier, France; 4 UMR 5535, Montpellier, France; 5 Rheumatology department, CHU de la Cavale Blanche & EA 2216, Brest, France; 6 Rheumatology Department & INSERM U699, APHP, Bichat Hospital, Paris, France; 7 Rheumatology Department, Lapeyronie Hospital, 371, Avenue du Doyen Gaston Giraud, 34295 Montpellier, Cedex 5, France

**Keywords:** Rheumatoid arthritis, Early arthritis, Seronegative arthritis, Arthritis, ESPOIR cohort, Anti-citrullinated protein antibodies (ACPA), Rheumatoid factor

## Abstract

**Objective:**

To describe the disease course of patients with early arthritis without rheumatoid factor (RF) and anti-citrullinated protein auto-antibodies (ACPA) in an inception cohort. To determine baseline predictors of fulfilling 2010 ACR/EULAR criteria for rheumatoid arthritis (RA) for these patients within 3 years.

**Method:**

Patients included in the multicenter ESPOIR cohort were compared at baseline and 3 years by whether they were negative for RF and ACPA (“seronegative”) or positive for RF and/or ACPA (“seropositive”). Univariate analysis was used to determine the association between baseline variables in seronegative patients and RA classification. Stepwise multiple logistic regression was used to identify predictors of RA classification within 3 years, estimating odds ratios (ORs).

**Results:**

Among 354 seronegative patients, 224/340 with available data (65.9%) fulfilled RA classification at baseline and 189/233 (81.1%) at 3 years. As compared with seropositive patients, seronegative patients had lower DAS28 (*p* = 0.002) and lower modified total Sharp score (mTSS; *p* = 0.026) at baseline; DAS28 remission was similar (*p* = 0.634), but radiographic progression rate was lower in seronegative patients (*p* <  0.001) at 3 years. In seronegative patients, factors predicting RA classification within 3 years were additive (OR = 3.61), bilateral (OR = 2.59) and hand, wrist or forefeet involvement (OR = 3.87); presence of a trigger event (OR = 3.57); pain at rest (OR = 2.76); morning stiffness (OR = 2.62); number of tender joints (OR = 23.73); and mTSS (OR = 2.56).

**Conclusion:**

“Seronegative” patients have less active disease at baseline and less radiographic progression during follow-up than “seropositive” patients. With inflammatory pain, symmetric involvement of numerous small joints and erosive disease, a classification of RA is likely.

**Electronic supplementary material:**

The online version of this article (10.1186/s13075-019-1909-8) contains supplementary material, which is available to authorized users.

## Background

Diagnosing rheumatoid arthritis (RA) at an early stage remains a challenge for clinicians. Indeed, no test is sufficiently specific to identify RA with certainty. The diagnosis is based on a spectrum of clinical, biological, and radiographic features, although the latter, despite a high specificity when typical erosions are observed, are rarely present at an early stage of the disease. Thus, testing for auto-antibodies such as rheumatoid factor (RF) and anti-citrullinated protein antibodies (ACPA) is useful to diagnose RA among patients with early arthritis [1].

ACPA such as anti-cyclic-citrullinated peptide (anti-CCP) antibodies are highly specific to RA [2, 3], have good predictive validity for RA in patients with early arthritis [4], and are associated with radiographic progression in early RA [5, 6]. Although RF is considered less specific [3], it is also associated with worst radiographic outcome [7, 8]. ACPA and RF have the same weight in the 2010 American College of Rheumatology/European League Against Rheumatism (ACR/EULAR) classification criteria for RA [9] and have a major impact in the diagnosis and prognosis of RA. In clinical practice, the decision to start a disease-modifying anti-rheumatic drug (DMARD) is more difficult in the absence of RF and ACPA [10] since these auto-antibodies are important features both for diagnosis and for risk of persistency and erosiveness. Nevertheless, 20 to 30% of RA patients do not have ACPA [2] or RF, and erosive RA may exist without these two auto-antibodies.

Distinct genetic risk factors are associated with ACPA-positive or ACPA-negative disease. Anti-CCP–positive RA was found associated with HLA-DRB1, HLA-DP, PTPN22, C5-TRAF1, and TNFAIP3-OLIG3 polymorphisms [11–13], whereas anti-CCP–negative RA was found associated with genes such as HLA-DR3 and IRF-5 [13, 14], two genes that are also associated with systemic lupus erythematosus and Sjögren syndrome. These data might indicate distinct pathogenic mechanisms underlying ACPA-positive and ACPA-negative RA, with the last entity not well defined. Whether ACPA-negative RA features auto-antibodies binding to other citrullinated proteins (vimentin etc.) not detected in routine care or whether it is true RA without auto-antibodies is unknown. Because the clinical presentation of early arthritis is not specific, we do not know whether RA without ACPA or RF is “true” RA or another undifferentiated inflammatory arthritis. The literature contains few data specifically regarding the follow-up of “seronegative” early arthritis (i.e., negative for RF and ACPA).

The objectives of this study were first to describe the disease course of patients without RF and ACPA in an inception cohort of early inflammatory arthritis patients and second to determine baseline predictors of fulfilling 2010 ACR/EULAR criteria for RA within 3 years in these patients.

## Patients and methods

### Study population

The ESPOIR cohort included 813 patients with early arthritis from 14 French rheumatology centers between 2002 and 2005 [15]. Patients were eligible if they had a definitive or probable clinical diagnosis of RA or polyarthritis not better explained by another etiology; had two or more swollen joints for more than 6 weeks and less than 6 months; and did not receive DMARDs or steroids for more than 2 weeks, and if administered, steroids were stopped at least 2 weeks before inclusion. We excluded patients with a definite diagnosis different from RA. Patients were evaluated every 6 months for 2 years and then once a year and were cared for as routine by their rheumatologist. The protocol of the ESPOIR cohort was approved by the ethics committee of Montpellier, France (no. 020307, CNIL 02-1293), and all patients gave their signed informed consent before inclusion.

### Auto-antibodies

Patients were tested for RF and anti-CCP antibodies. Anti-CCP antibodies were analyzed by Elecsys assay (Roche Diagnostics, Switzerland), with titers > 17 U/ml considered positive. IgM-RF was analyzed by using an ELISA kit (Ménarini, France), with titers > 9 IU/ml considered positive. The analyses of RF and anti-CCP status were centralized and performed in the Department of Immunology, Bichat University Hospital, Paris.

### Baseline assessment

The following data were collected at baseline and at each visit: demographic data, comorbidities, current tobacco use, current alcohol consumption, family history of RA, duration of symptoms at first visit (defined by the date of the first fixed swollen joint), presence of a trigger event (death of a relative/loved one, trauma, vaccination, hormonal medication, delivery), clinical features of arthritis (duration of morning stiffness, pain at rest and on mobility on a 0–100 mm visual analog scale), number of tender and swollen joints in 28 joints, initial joint topography (additive, bilateral, distal (hands, wrists or forefeet), or proximal (elbows, shoulders, ankles, knees) involvement), extra-articular manifestations (Sicca syndrome, nodules, Raynaud syndrome), Disease Activity Score in 28 joints–erythrocyte sedimentation rate (DAS28-ESR), functional disability evaluated by the Stanford Health Assessment Questionnaire Disability Index (HAQ-DI), biological features (including ESR, [mm/h], C-reactive protein [CRP; mg/l] level by standard laboratory methods, auto-antibodies previously described, anti-nuclear antibodies (ANA), HLA-DRB1* genotype), and radiographs of hands, wrists, and forefeet in the posteroanterior view.

### Follow-up assessment

All patients were followed up for 3 years. A clinical evaluation and blood test for acute phase reactants were performed every year using the technique previously described. RF and anti-CCP antibodies were tested at each visit. Treatments (conventional synthetic [cs] or biological [b] DMARDs, steroids) were reported. Radiographs were obtained every year by using the same technique.

### Radiographic evaluation

Radiographs were stored in the Department of Rheumatology, Brest Hospital (Brest, France), and blindly scored for presence of erosions and joint space narrowing according to the van der Heijde-modified total Sharp score (mTSS) [16] by an experienced imaging reader (CL) who was blinded to patients’ other data. Radiographs were scored in a chronological order. Typical RA erosion was defined as previously described [17]. Radiographic progression was defined by an increase of at least 1 unit in the mTSS.

### Outcome

We considered a diagnosis of RA as the ability of each patient to fulfill the 2010 ACR/EULAR classification criteria [9]. The patients were classified as RA or not according to the 2010 ACR/EULAR criteria at any visit during the first 3 years of follow-up. Alternative diagnoses were reported among patients who did not fulfill the 2010 ACR/EULAR criteria.

### Statistical analysis

Descriptive statistics are presented as mean (SD), median (IQR), or number (%) as appropriate. Comparisons between patients with RF and/or ACPA positivity and without RF and ACPA positivity (as defined above) were performed at baseline and at 3 years by chi-square test or Fisher’s exact test as appropriate for qualitative variables and Wilcoxon test for quantitative variables. Similarly, comparisons between all baseline values and outcome measures involved use of the chi-square test or Fisher’s exact test for categorical data and Wilcoxon test for continuous variables. For the multivariate analysis, continuous variables were transformed into categorical variables with the median or a predetermined threshold used as the cut-off. The explanatory variables included in the multiple regression model had *p* <  0.20 on univariate analysis. Stepwise logistic regression analysis was used to determine relevant independent baseline variables to predict RA classification during the first 3 years. Significance was defined as *p* <  0.05 for variables in the multivariate model. SAS v9.4 was used for analysis (SAS Institute, Cary, NC, USA).

## Results

### Baseline characteristics

Among the 813 included patients, serology data were missing for one patient and 64 were excluded because of another definite diagnosis than RA or undifferentiated arthritis finally made during the 3 years of follow-up: psoriatic arthritis (*n* = 11), spondyloarthritis (*n* = 10), osteoarthritis (*n* = 9), connective tissue disease and vasculitis (*n* = 16), polymyalgia rheumatica and RS3PE syndrome (*n* = 3), fibromyalgia (*n* = 6), or other (*n* = 9).

**Fig. 1:**
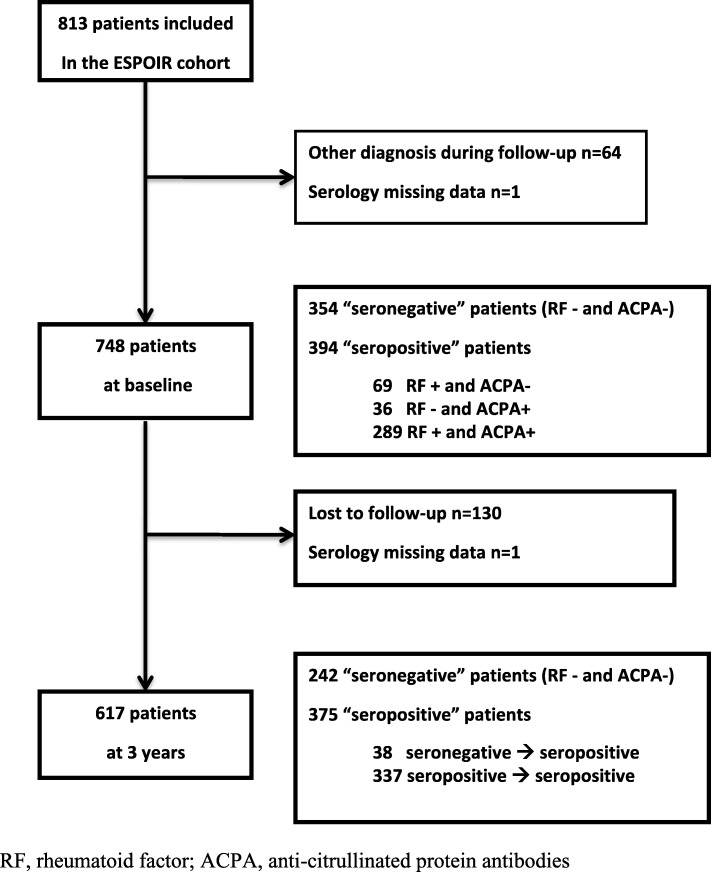
Flow-chart of the patients included in the study. RF, rheumatoid factor; ACPA, anti-citrullinated protein antibodies

Among the 748 remaining patients, 354 (47.3%) were negative for both IgM-RF and anti-CCP2 antibodies and 394 (52.7%) were positive for IgM-RF and/or anti-CCP2 antibodies. The flow-chart of patients is presented in Fig. 1**.** The baseline characteristics of the 748 patients are in Table 1**.** Seronegative patients had less active disease than seropositive patients (mean DAS28-ESR 5.0 [SD 1.3] vs 5.3 [SD 1.3], *p* = 0.002) and had slightly less structural damage (mean mTSS 4.3 [6] vs 5.7 [8.2], *p* = 0.026) and less disability (mean HAQ-DI 0.9 [0.7] vs 1 [0.7], *p* = 0.030). HLA-DRΒ1*01 or 04 gene was less frequent in seronegative than seropositive patients (31.6% vs 61.2%, *p* <  0.001), and the opposite was found for HLA-DRB1*03 (21.7% vs 15.2%, *p* = 0.021). ANA were more frequent in seropositive than seronegative patients (28.5% vs 12.8%, *p* <  0.001). Extra-articular manifestations were similar in both groups **(**Table 1)**.**

**Table 1:** Baseline characteristics of patients with “seronegative” and “seropositive” early arthritis (*n* = 748)

	Seronegative (*n* = 354)	Seropositive (*n* = 394)	*P* value**
Female, *n* (%)	274 (77.4%)	305 (77.4%)	0.997
Age (years), mean (SD)	48.9 (13.2)	47.7 (11.9)	0.058
Caucasian, *n* (%)	330 (93.2%)	359 (91.1%)	0.287
Current tobacco exposure, *n* (%)	173 (48.9%)	187 (47.5%)	0.700
Current alcohol consumption, *n* (%)	61 (17.2%)	71 (18%)	0.778
Symptom duration at first visit (months), mean (SD)	3.4 (1.7)	3.4 (1.8)	0.693
Morning stiffness (min), mean (SD)	75.6 (144.6)	110.1 (214.8)	*0.008*
Pain at rest (VAS, 0–100), mean (SD)	37.2/100 (27.6)	38/100 (27.5)	0.684
TJC, mean (SD)	8.8 (7.2)	8.3 (6.9)	0.521
SJC, mean (SD)	7.1 (5.5)	7.6 (5.4)	0.077
Extra-articular manifestations ♯	188/346 (54.3%)	191/383 (52.5%)	0.228
Anti-CCP positive, *n* (%)	0	325 (82.5%)	–
Anti-CCP, units/ml mean (SD)	0.2 (1.3)	292.3 (201.7)	*< 0.001*
IgM-RF positive, *n* (%)	0	358 (90.9%)	–
IgM-RF, IU/ml, mean (SD)	4.6 (1.5)	238.5 (770.8)	*< 0.001*
ANA positive, *n* (%)	45 (12.8%)	111 (28.5%)	*< 0.001*
ESR, mm/h, mean (SD)	25.8 (23.3)	32.9 (24.8)	*< 0.001*
CRP level, mg/L mean (SD)	18.3 (33.1)	21.9 (30.6)	*< 0.001*
DAS28, mean (SD)	5.0 (1.3)	5.3 (1.3)	*0.002*
HAQ-DI, mean (SD)	0.9 (0.7)	1 (0.7)	*0.030*
mTSS, mean (SD)	4.3 (6)	5.7 (8.2)	*0.026*
HLA-DRΒ1*01 or 04 gene, *n* (%)	112 (31.6%)	241 (61.2%)	*< 0.001*
HLA-DRΒ1*03 gene, *n* (%)	77 (21.8%)	60 (15.2%)	*0.021*
2010 ACR/EULAR criteria (≥ 6/10 points), *n* (%)	224/340 (65.9%)	386/394 (98.0%)	*< 0.001*
Based on score ≥ 6	206/353 (58.4%)	384/394 (97.5%)	*< 0.001*
Typical RA erosion	60/318 (18.9%)	113/362 (31.2%)	*< 0.001*

“seronegative” early arthritis: patients negative for RF and ACPA; “seropositive” early arthritis: patients positive for RF and/or ACPA

**Pearson’s chi-square test or Fisher’s exact test for categorical data and Wilcoxon test for continuous variables

♯ Nodules, dry eye, and dry mouth, salivary gland swelling, Raynaud syndrome, purpura, adenopathy, splenomegaly, pericarditis, pleural effusion, polyneuropathy, scleritis, keratitis, uveitis, carpal tunnel syndrome

*VAS* visual analog scale, *TJC* tender joint count, *SJC* swollen joint count, *anti-CCP* anti-cyclic-citrullinated peptide, *RF* rheumatoid factor, *ANA* anti-nuclear antibodies, *ESR* erythrocyte sedimentation rate, *CRP* C-reactive protein, *DAS28* Disease Activity Score in 28 joints, *HAQ-DI* Health Assessment Questionnaire Disability Index, *mTSS* modified total Sharp score, *ACR/EULAR* American College of Rheumatology/European League Against Rheumatism, *RA* rheumatoid arthritis

*P* values were checkedEntries in italics were significant

Overall, 224/340 (65.9%) seronegative patients with all available data fulfilled 2010 ACR/EULAR criteria for RA at baseline as compared with 386/394 (98%) seropositive patients (*p* <  0.001). Among them, 60/318 (18.9%) seronegative patients had typical RA erosions [17] as compared with 113/362 (31.2%) seropositive patients (*p* <  0.001). Other baseline characteristics did not differ between the two groups.

### RA outcome at 3 years

Data were available for 617 (242 seronegative and 375 seropositive) patients followed up at 3 years (Table 2). A total of 38 patients who were seronegative at baseline showed RF (*n* = 30), anti-CCP2 (*n* = 7), or both auto-antibodies (*n* = 1) during the 3 years of follow-up and were considered seropositive. Baseline characteristics of these 617 patients were similar to the whole cohort. Baseline HAQ-DI and mTSS data were numerically lower for seronegative compared to seropositive patients reaching 3 years follow-up, but did not reach significance (Additional file 1: Table S1), whereas the difference was significant when considering the whole population (Table 1), probably due to a higher sample size. Finally, 189/233 (81.1%) seronegative and 369/371 (99.4%) seropositive patients with data available were classified as having RA during the 3 years of follow-up (*p* <  0.001); 71 (29.3%) and 235 (62.7%) had typical RA erosions (*p* <  0.001) (Table 2).

**Table 2:** Characteristics of patients with “seronegative” and “seropositive” early arthritis at 3 years (*n* = 617)

	Seronegative (*n* = 242)	Seropositive (*n* = 375)	*P* value*
TJC, mean (SD)	3 (5.3)	2.9 (5.3)	0.900
SJC, mean (SD)	1 (2)	1.5 (2.7)	*0.034*
ESR, mm/h, mean (SD)	12.4 (10.4)	16.6 (14.7)	*< 0.001*
CRP, mg/L mean (SD)	6 (11)	7.3 (11.6)	*0.025*
DAS28, mean (SD)	2.8 (1.3)	2.9 (1.4)	0.211
DAS28 < 2.6, *n* (%)	115 (49.4%)	170 (47.4%)	0.634
HAQ-DI, mean (SD)	0.5 (0.6)	0.5 (0.6)	0.980
mTSS, mean (SD)	10.7 (12.1)	16.3 (15.8)	*< 0.001*
Δ mTSS versus baseline, mean (SD)	5.8 (7.9)	10.4 (11.3)	*< 0.001*
2010 ACR/EULAR criteria (≥ 6/7 points), *n* (%)	189/233 (81.1%)	369/371 (99.4%)	*< 0.001*
Based on score ≥ 6	166/229 (72.5%)	366/371 (98.7%)	*< 0.001*
Typical RA erosion	71/242 (29.3%)	235/375 (62.7%)	*< 0.001*
cs and b DMARDs, *n* (%)	134 (55.6%)	302 (80.8%)	*< 0.001*
cs DMARDs, *n* (%)	133 (55.2%)	290 (77.5%)	*< 0.001*
b DMARDs, *n* (%)	10 (4.2%)	67 (17.9%)	*< 0.001*
Corticosteroids, *n* (%)	59 (24.5%)	130 (34.8%)	*0.007*

*Pearson’s chi-square test or Fisher’s exact test for categorical data and Wilcoxon test for continuous variables

*TJC* tender joint count, *SJC* swollen joint count, *ESR* erythrocyte sedimentation rate, *CRP* C-reactive protein, *DAS28* Disease Activity Score in 28 joints, *HAQ-DI* Health Assessment Questionnaire Disability Index, *mTSS* modified total Sharp score, *ACR/EULAR* American College of Rheumatology/European League Against Rheumatism, *RA* rheumatoid arthritis, *cs* conventional synthetic, *b* biological, *DMARD* disease-modifying anti-rheumatic drugs

*P* values were checkedEntries in italics were significant

Seronegative patients had similar mean DAS28-ESR and mean HAQ-DI as seropositive patients (Table 2). The proportion of patients achieving DAS28 remission was similar. The mean mTSS and radiographic progression at 3 years were lower in the seronegative group. These patients also less frequently had csDMARDs or bDMARDs or used corticosteroids than seropositive patients.

The 38 patients who switched from seronegative to seropositive did not show a significantly different outcome as compared with patients who were seropositive at baseline (data not shown).

### Predictors of RA classification within 3 years

On univariate analysis, RA classification by 3 years was significantly associated with several baseline parameters among seronegative patients (Table 3). In particular, at baseline, patients fulfilling 2010 ACR/EULAR criteria for RA within 3 years had higher values than those without an RA classification for the following: median (IQR) number of tender joints (9 [5–16] vs 2 [0–4], *p* <  0.001) and swollen joints (7 [4–12] vs 3 [2–5], *p* <  0.001), and mean (SD) DAS28 (5.3 [1.2] vs 3.8 [1.1], *p* <  0.001), HAQ-DI (1 [0.7] vs 0.6 [0.6], *p* = 0.008), and mTSS (5 [6.6] vs 2.4 [3.1], *p* = 0.008). A trigger event (*p* = 0.004), in particular the death of a relative or loved one (*p* = 0.0253), was more frequent in patients with than without an RA classification during the follow-up. However, some other factors such as presence of anti-nuclear antibodies or harboring HLA-DRΒ1*03 were less frequent in patients with than without RA although not significantly. Mean ESR or CRP level was not significantly higher in RA patients.

**Table 3:** Univariate and multivariate logistic regression for fulfillment of 2010 ACR/EULAR classification criteria within 3 years in seronegative patients

Baseline variables	RA classification		Univariate analysis		Multivariate analysis^a^ AUC=0.905	
	Yes 267/323 (82.7%)	No 56/323 (17.3%)	OR (95% CI)	p value*	OR (95% CI)	*p* value*
Sex (female vs male)	213 (79.8%)	39 (69.6%)	0.58 [0.31; 1.11]	0.096		
Age *Median>52 years*	144 (53.9%)	25 (44.6%)	1.45 [0.81; 2.59]	0.206		
Menopause (n=252)	116 (54.5%)	14 (35.9%)	2.14 [1.05; 4.33]	*0.033*		
Baseline BMI *Median>24.5 kg/m* ^*2*^	133 (50%)	25 (44.6%)	1.24 [0.69; 2.21]	0.466		
Family history of RA	38 (14.2%)	3 (5.4%)	2.93 [0.87; 9.86]	0.070**		
Trigger event	90 (33.7%)	8 (14.3%)	3.05 [1.38; 6.72]	*0.004*	3.57 [1.33; 9.60]	*0.012*
Bilateral involvement	240 (89.9%)	27 (48.2%)	9.55 [4.94; 18.44]	*<0.001*	2.59 [1.14; 5.90]	*0.023*
Distal involvement	258 (96.6%)	41 (73.2%)	10.49 [4.31; 25.53]	*<0.001*	3.87 [1.25; 11.93]	*0.019*
Proximal involvement	146 (54.7%)	16 (28.6%)	3.02 [1.61; 5.65]	*<0.001*		
Additive involvement	52 (19.5%)	6 (10.7%)	2.02 [0.82; 4.95]	0.120	3.61 [1.09; 12.02]	*0.036*
Initial presentation:						
-acute	39 (14.6%)	9 (16.1%)				
-subacute	76 (28.5%)	22 (39.3%)	-	*0.021*		
-incidious	152 (56.9%)	25 (44.6%)				
Extra-articular manifestation	148 (56.9%)	27 (49.1%)	1.37 [0.77; 2.45]	0.288		
Sicca syndrome	89 (33.3%)	13 (23.2%)	1.65 [0.85; 3.23]	0.139		
Family history of psoriasis	36 (13.5%)	2 (3.6%)	4.21 [0.98; 18.02]	0.036**		
Psoriasis	18 (6.7%)	2 (3.6%)	1.95 [0.44 ; 8.66]	0.371**		
Pain at rest (VAS, 0-100) *Median>34*	150 (56.2%)	15 (26.8%)	3.50 [1.85; 6.64]	*<0.001*	2.76 [1.25; 6.11]	*0.012*
Pain on mobility (VAS, 0-100) *Median>55*	145 (54.3%)	16 (28.6%)	2.97 [1.59; 5.57]	*<0.001*		
Morning stiffness duration *Median> 38 min*	157 (58.8%)	19 (33.9%)	2.78 [1.52; 5.09]	*<0.001*	2.62 [1.20; 5.69]	*0.015*
TJC *Median>7*	155 (58%)	2 (3.6%)	37.37 [8.92; 156.5]	<0.001**	23.73 [5.25; 107.33]	*<0.001*
SJC *Median>6*	163 (61%)	11 (19.6%)	6.41 [3.17; 12.96]	*<0.001*		
DAS28 *Median >4.9*	164 (62.1%)	11 (19.6%)	6.71 [3.32; 13.57]	*<0.001*		
ANA Positive if >1/160	31(11.6%)	9 (16.1%)	0.69 [0.31; 1.55]	0.368		
HAQ >0.9	133 (49.8%)	17 (30.4%)	2.28 [1.23; 4.22]	*0.008*		
IgA-RF	30 (11.2%)	3 (5.4%)	2.24 [0.66; 7.60]	0.187		
HLA-DRΒ1*03 gene	54 (20.2%)	15 (26.8%)	0.69 [0.36; 1.34]	0.276		
mTSS *Median >2*	132 (51.6%)	18 (32.1%)	2.25 [1.22; 4.14]	*0.008*	2.56 [1.18; 5.56]	*0.018*
Typical RA erosion [17]	60 (24.8%)	0	-	*<0.001*		

* chi-square test **Fisher’s exact test

^a^Included variables: sex, initial presentation, initial trigger event, morning stiffness, pain at rest and at mobility, number of tender and swollen joints, HAQ, feet erosion, wrists and hands erosion, bilateral, proximal or distal involvement, additive involvement, sicca syndrome

*AUC* area under the receiver operating characteristic curve, *BMI* body mass index, *VAS* visual analog scale, *TJC* tender joint count, *SJC* swollen joint count, *DAS28* Disease Activity Score in 28 joints, *ANA* antinuclear antibodies, *RF* rheumatoid arthritis, *mTSS* modified total Sharp score, *RA* rheumatoid arthritis

*P* values were checkedEntries in italics were significant

Stepwise logistic regression analysis showed that in the seronegative cohort, RA classification within 3 years was associated with the baseline factors additive, bilateral, and distal (i.e., hands, wrists, or forefeet) involvement; presence of a trigger event; pain at rest; morning stiffness; number of tender joints; and mTSS (Table 3), with no association between extra-articular manifestations, harboring HLA- DRΒ1*03 and RA classification.

## Discussion

When patients present inflammatory arthritis, physicians must identify disease that will progress to RA. Because auto-antibodies such as RF or ACPA are key in the diagnosis, their weight is important (up to 3 of 10 points) in the 2010 ACR/EULAR classification criteria for RA. Thus, obtaining 6 of 10 points required for such a classification of RA in the absence of these 2 auto-antibodies may be difficult in the early phases of the disease, since it requires the involvement of more than 10 involved joints. Although this situation is frequently encountered, especially in early RA, the initial clinical presentation and disease course of seronegative RA is not well known. We first compared the initial features of patients with early arthritis according to positivity for RF and/or ACPA. We used a large, prospective, early-arthritis cohort from the community. This situation reflects clinical practice and allowed us to study the clinical value of RF and ACPA in patients selected by symptoms, not diagnosis. The patients in our 2 seronegative and seropositive groups were well balanced, thus confirming that almost half of the patients were negative for RF and ACPA at inclusion in this primary care–based cohort. The disease was less active based on DAS28-ESR and also less severe in terms of functional index and radiographic score at baseline in seronegative versus seropositive group. These results agree with those of the Norfolk Arthritis Register (NOAR) [18, 19] but not with the Canadian early-arthritis cohort (CATCH), showing seronegative patients with higher mean swollen joint count, DAS28, and erosive disease [20], which suggests that these patients are more frequently referred to rheumatology if they have more active and severe disease. The disease progression was less severe and DMARD or steroid use less frequent in seronegative versus seropositive group during follow-up in the ESPOIR cohort, which agrees with other early-arthritis cohorts [18, 20]. In our cohort, about 10% of patients developed de novo auto-antibodies (RF or anti-CCP2) during the 3 years of follow-up. This proportion was less in another Dutch early-arthritis cohort, in which the switch from negative to positive occurred in 2% for ACPA and 3% for IgM-RF during the first year of follow-up [21].

The secondary objective of our study was to determine baseline predictors of fulfilling the 2010 ACR/EULAR criteria for RA within 3 years in these patients. We were looking for factors other than auto-antibodies that suggest the development of RA. The best independent predictive factors were additive, bilateral, and distal involvement; presence of a trigger event; pain at rest; morning stiffness; number of tender joints; and mTSS. In the prediction model for persistent erosive arthritis published by Visser et al., similar baseline variables were selected, including morning stiffness for at least 1 h, arthritis in at least three joint groups, IgM-RF, and anti-CCP2 positivity, in addition to erosions on hand and foot radiographs that were not selected in our model [22].

ACR/EULAR classification criteria were used to guide the diagnosis of RA, which may lead to incorporation bias, because explanatory variables were part of the reference standard. This observation may suggest overestimation of the discriminative ability of the model [23]. To partly solve this problem, we could have added expert opinion to the ACR/EULAR criteria, although incorporation bias still exists in this case. This bias can explain the important weight of the number of tender joints in the model. Of note, the area under the receiver operating characteristic curve for the stepwise logistic regression analyses was high (0.905), which suggests that these predictors are efficient to differentiate early in the disease between RA and other forms of arthritis in clinical practice. ACR/EULAR classification criteria were built based on cohorts of real-world patients with early arthritis, to identify factors and their relative weights, which were associated with the subsequent decision by a physician to start methotrexate. Consequently, they were better adapted to the classification of patients with early arthritis. Nevertheless, performance of these criteria have also been studied in patients with established disease and had the same sensitivity, but higher specificity than the 1987 ACR criteria for predicting a diagnosis of RA after 10 years, mainly due to the use of exclusion criteria [24].

Nevertheless, some limitations in our study should be noted. First, patients were defined as seronegative if they did not have IgM-RF and anti-CCP2 antibodies. Some seronegative patients may have had other RA-associated antibodies, such as IgA-RF or anticarbamylated antibodies, which were not tested in our study. However, based on currently available studies, these patients likely represent a small proportion of the seronegative population [25]. Among 318 patients with anti-CCP-negative RA (1987 ACR criteria) included in the Leiden early-arthritis cohort, a cluster analysis performed to evaluate whether patients resemble each other revealed no grouping of patients, which suggests that anti-CCP–negative RA patients may be homogeneous [26]. Second, more patients were lost to follow-up in the seronegative group, even after accounting for the 38 patients with auto-antibodies during follow-up. This finding can be explained by patients with severe RA more likely to be followed up. Previous data noted that anti-CCP antibodies were the best predictor of remaining in the ESPOIR cohort for 5 years [27].

## Conclusion

Patients with early arthritis and without RF and ACPA have less active disease at baseline and less severe disease during follow-up. They are more likely to fulfill RA classification at 3 years if they have RA typical inflammatory characteristics of pain, symmetric involvement of numerous small joints, and initial erosive disease.

## Additional file

Additional file 1: **Table S1.** Baseline characteristics of patients with “seronegative” and “seropositive” early arthritis with data available at 3 years (*n* = 617). (DOCX 115 kb)

## Abbreviations

ACPA: Anti-citrullinated protein antibodies
ACR: American College of Rheumatology
ANA: Anti-nuclear antibodies
Anti-CCP: Anti-cyclic-citrullinated peptide
APHP: Assistance publique – hôpitaux de Paris
b DMARD: Biological DMARD
CATCH: Canadian early-arthritis cohort
CHU: Centre hospitalier universitaire
CNIL: Commission nationale de l'informatique et des libertés
CRP: C-reactive protein
cs DMARD: Conventional synthetic DMARD
DAS28: Disease activity score in 28 joints
DMARD: Disease-modifying anti-rheumatic drug
EA: équipe d’accueil
ESPOIR: Etude et Suivi des POlyarthrites Indifferenciées Récentes
ESR: Erythrocyte sedimentation rate
EULAR: European League Against Rheumatism
HAQ-DI: Stanford Health Assessment Questionnaire Disability Index
HLA: Human leucocyte antigen
Ig: Immunoglobulin
INSERM: Institut national de la santé et de la recherche médicale
IQR: Interquartile range
IRF-5: Interferon regulatory factor 5
mTSS: Modified total Sharp score
NOAR: Norfolk Arthritis Register
ORs: Odds ratios
PTPN22: Protein tyrosine phosphatase, non receptor type 22
RA: Rheumatoid arthritis
RF: Rheumatoid factor
RS3PE syndrome: Remitting seronegative symmetrical synovitis with pitting edema
SAS: Statistical Analysis System
SD: Standard deviation
TNFAIP3-OLIG3: Tumor necrosis factor Alpha Induced Protein 3 - oligodendrocyte transcription factor 3
TRAF1/C5: Tumor necrosis factor-receptor associated factor 1/complement component 5
UMR: unité mixte de recherche
Univ: University
Vs: Versus
