# Supplementary material for: Outcome of patients with early arthritis without rheumatoid factor and ACPA and predictors of rheumatoid arthritis in the ESPOIR cohort
Source: Arthritis Res Ther. 2019 Jun 6;21:140. doi: 10.1186/s13075-019-1909-8 (PMC6555707; doi:10.1186/s13075-019-1909-8)
Supplement: Supplementary file 1 — Table S1. Baseline characteristics of patients with “seronegative” and “seropositive” early arthritis with data available at 3 years (n = 617). (DOCX 115 kb) [file 13075_2019_1909_MOESM1_ESM.docx]

***Supplementary Table 1. Baseline characteristics of patients with “seronegative” and “seropositive” early arthritis with data available at 3 years (n=617).***

|  | **Seronegative**  **(n=280)** | **Seropositive**  **(n=337)** | **P value*** |
| --- | --- | --- | --- |
| Female, n (%) | 215 (76.8%) | 259 (76.8%) | 0.984 |
| Age (years), mean (SD) | 49.9 (12.5) | 47.8 (11.5) | **0.009** |
| Caucasian, n (%) | 265 (94.6%) | 311 (92.3%) | 0.242 |
| Tobacco exposure, n (%) | 129 (46.1%) | 162 (48.1%) | 0.620 |
| Alcohol consumption, n (%) | 52 (18.6%) | 65 (19.3%) | 0.821 |
| Symptom duration at first visit (months), mean (SD) | 3.3 (1.6) | 3.4 (1.8) | 0.550 |
| Morning stiffness (min), mean (SD) | 80.6 (158.8) | 112.5 (228.6) | 0.058 |
| Pain at rest (VAS, 0-100), median (IQR) | 37/100 (13–57.5) | 36.6/100 (10–53) | 0.973 |
| TJC, median (IQR) | 8.7 (3–13.5) | 8.1 (3–12) | 0.436 |
| SJC, median (IQR) | 7.5 (3–10) | 7.5 (3–11) | 0.824 |
| Anti-CCP–positive, n (%) | - | 280 (83.1%) | - |
| Anti-CCP, units/ml mean (SD) | 0.2 (1.4) | 300.8 (201.8) | **<0.001** |
| IgM-RF–positive, n (%) | - | 304 (90.2%) | - |
| IgM-RF, IU/ml, mean (SD) | 4.6 (1.6) | 209.5 (578.3) | **<0.001** |
| ANA-positive, n (%) | 31 (11.1%) | 90 (27%) | **<0.001** |
| ESR, mm/h, mean (SD) | 25.7 (23.5) | 32.2 (24.7) | **<0.001** |
| CRP level, mg/L mean (SD) | 18.9 (34.9) | 21.3 (31.3) | **0.010** |
| DAS28, mean (SD) | 5.0 (1.3) | 5.2 (1.3) | **0.026** |
| HAQ-DI, mean (SD) | 0.9 (0.7) | 1 (0.7) | 0.086 |
| mTSS, mean (SD) | 4.6 (6.2) | 6.1 (8.6) | 0.067 |
| HLA-DRΒ1*01 or 04 gene, n (%) | 92 (32.9%) | 210 (62.3%) | **<0.001** |
| HLA-DRΒ1*03 gene, n (%) | 59 (21.1%) | 48 (14.2%) | **0.026** |
| 2010 ACR/EULAR criteria (6/7 points), n (%) | 184/279 (65.9%) | 330/337 (97.9%) | **< 0.001** |
| - Based on score ≥ 6 | 168/280 (60%) | 328 /337 (97.3%) | **< 0.001** |
| - Typical RA erosion | 52/273 (19%) | 103/322 (32%) | **< 0.001** |

* Pearson’s chi-square test or Fisher’s exact test for categorical data and Wilcoxon test for continuous variable

VAS, visual analog scale; TJC, tender joint count; SJC, swollen joint count; anti-CCP, anti-cyclic-citrullinated peptide; RF, rheumatoid factor; ANA, antinuclear antibodies; ESR, erythrocyte sedimentation rate; CRP, C-reactive protein; DAS28, Disease Activity Score in 28 joints; HAQ-DI, Health Assessment Questionnaire Disability Index; mTSS, modified total Sharp score; ACR/EULAR, American College of Rheumatology/European League Against Rheumatism; RA, rheumatoid arthritis
